# Supplementary material for: 3D chromatin interactions involving Drosophila insulators are infrequent but preferential and arise before TADs and transcription
Source: Nat Commun. 2023 Oct 21;14:6678. doi: 10.1038/s41467-023-42485-y (PMC10590426; doi:10.1038/s41467-023-42485-y)
Supplement: Supplementary file 1 — Supplementary Information [file 41467_2023_42485_MOESM1_ESM.pdf]

SUPPLEMENTARY INFORMATION RELATED TO

3D chromatin interactions involving *Drosophila* insulators are infrequent but preferential and arise before TADs and transcription

Olivier Messina[1], Flavien Raynal[2], Julian Gurgo[1], Jean-Bernard Fiche[1], Vera Pancaldi[2,3*], Marcelo Nollmann[1,*]

[1] *Centre de Biologie Structurale, Univ Montpellier, CNRS UMR 5048, INSERM U1054, Montpellier, France.*

[2] *Université de Toulouse, Inserm, CNRS, Université Toulouse III-Paul Sabatier, Centre de Recherches en Cancérologie de Toulouse, Toulouse, France.*

[3] *Barcelona Supercomputing Center, Barcelona, Spain.*

[*] Corresponding authors: vera.pancaldi@inserm.fr, marcelo.nollmann@cbs.cnrs.fr

Table of contents

Table of contents 2
Supplementary Figures 3
Supplementary Figure S1. Supplementary Data complementary to Figure 1. 4
Supplementary Figure S2. Supplementary Data complementary to Figure 2. 7
Supplementary Figure S3. Supplementary Data complementary to Figure 3. 9
Supplementary Figure S4. Supplementary Data complementary to Figure 4. 11
Supplementary Tables and Data 12
Supplementary References 12

Supplementary Figures

Supplementary Figure S1. Supplementary Data complementary to Figure 1.

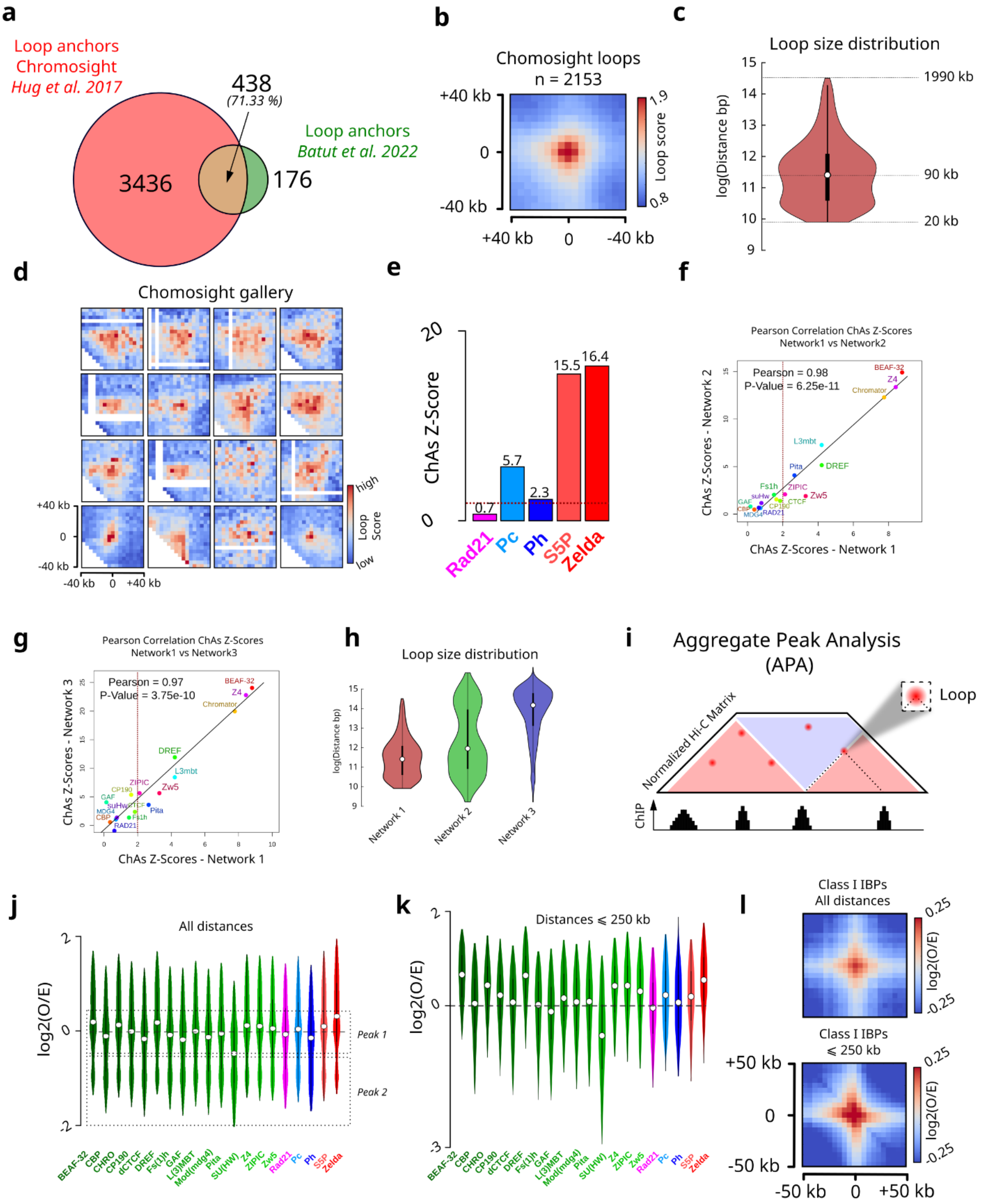

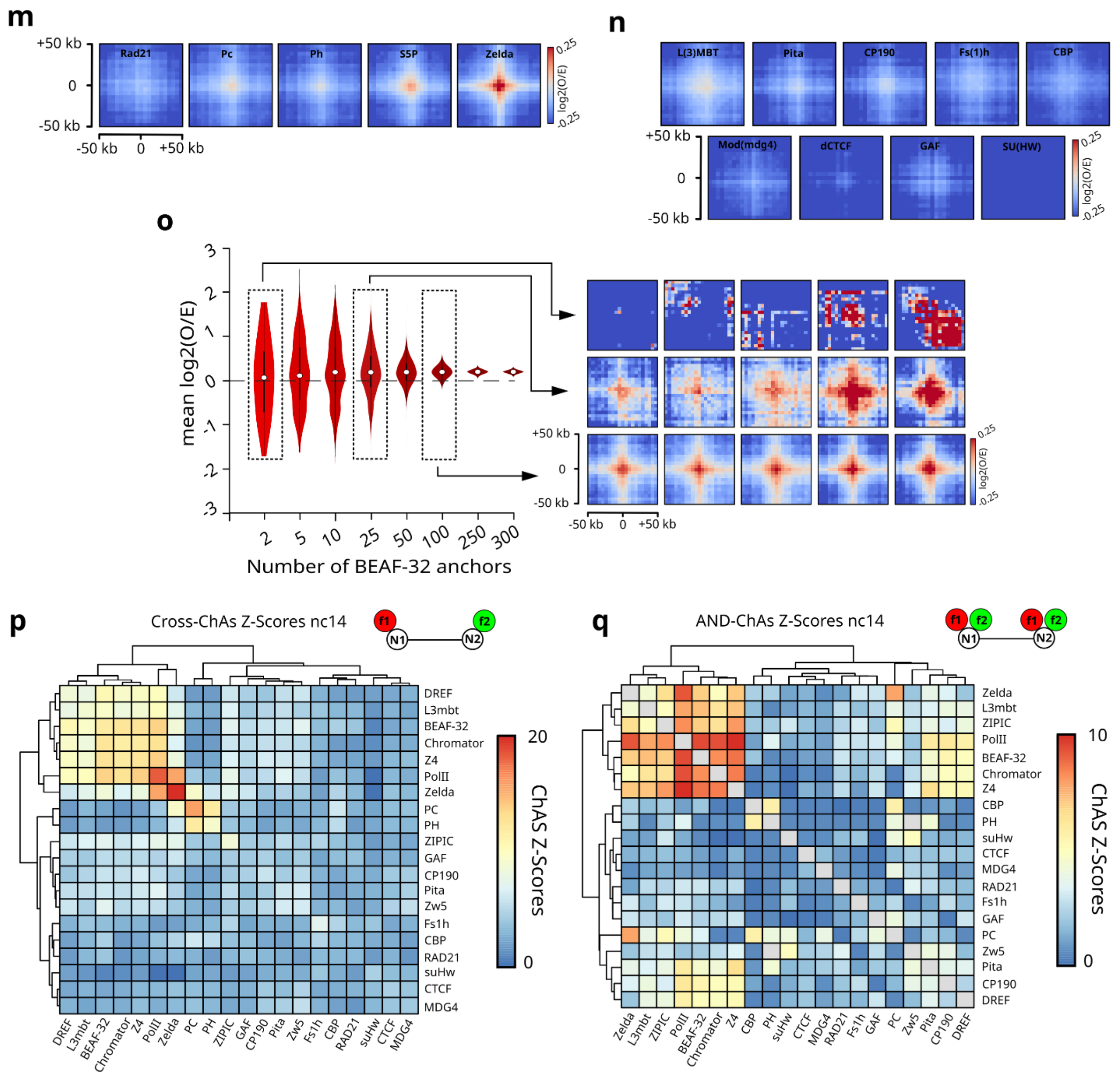

a. Venn diagram representing the overlap, at +/- 10kb, between loop anchors obtained from chromosight on data from Hug *et al.* [1] using the nc14 dataset, and loop anchors detected in *Batut et al.* 2022. [2] **b**. Pileup plot of all detected loops (2153) from Chromosight in nc14 embryos, centered on loop anchors, within a window of +/- 40kb. Red and blue represent high and low loop score values, respectively. **c**. Violin plot illustrating the loop size distribution from our Chromosight network 1. **d**. Examples of Hi-C maps showing regions with loops detected by Chromosight. Red and blue represent high and low loop scores, respectively. Matrices are distance normalized. **e**. Barplot illustrating the ChAs-Z-Scores for the cohesin subunit (Rad21), Polycomb group proteins (Pc, Ph), RNA polymerase II (RNAPII CTD phospho-Ser5) and the pioneering factors (Zelda) in the nc14 chromatin network classified by alphabetical order. **f**. Pearson correlation between ChAs Z-Scores of Network 1 and Network 2. (see *Methods*) **g.** Pearson correlation between ChAs Z-Scores of Network 1 and Network 3 (see *Methods*). **h.** Violin plots illustrating the loop size distribution from Chromosight network 1, 2 and 3. **i**. Cartoon illustrating Aggregate Peak Analysis (APA) of chromatin binding factors in distance normalized Hi-C matrix. **j**. Violin plots illustrating the distribution of the log2(O/E) for 15 IBPs, green, the cohesin subunit (Rad21), pink, Polycomb group proteins (Pc, Ph), blue, RNA polymerase II (RNAPII CTD phospho-Ser5) and the pioneering factors (Zelda), red, in the nc14 chromatin network for all genomic distances classified by

alphabetical order. Dashed rectangles pinpoint the two different peaks observed in the distribution. **k**. For genomic distances shorter than 250 kb. **l.** Aggregation Hi-C plots for nc14 embryos for Class I IBPs at all genomic distances (top) and when a maximum distance threshold of 250 kb is applied (bottom). **m**. Aggregation Hi-C plots for nc14 embryos for the cohesin subunit (Rad21), Polycomb group proteins (Pc, Ph), RNA polymerase II (RNAPII CTD phospho-Ser5) and the pioneering factors (Zelda), for all genomic distances. **n.** Aggregation Hi-C plots for nc14 embryos for Class II IBPs that do not display a positive log2(O/E) : L(3MBT), Pita, CP190, Fs(1)h, CBP, Mod(mdg4), dCTCF, GAF, SU(HW), for all genomic distances. **o**. Violin plots illustrating the distribution of the mean log2(O/E) for different sets of BEAF-32 anchors over 10000 iterations (left). Example of aggregation Hi-C plots for nc14 embryos for different numbers of BEAF-32 anchors (right) (see *Methods*). **p**. Heat map representing the ChAS Z-Scores from Cross-ChAs analysis on the nc14 chromatin network (see *Methods*). **q**. Heat map representing the ChAS Z-Scores from AND-ChAs analysis on the nc14 chromatin network (see *Methods*).

Supplementary Figure S2. Supplementary Data complementary to Figure 2.

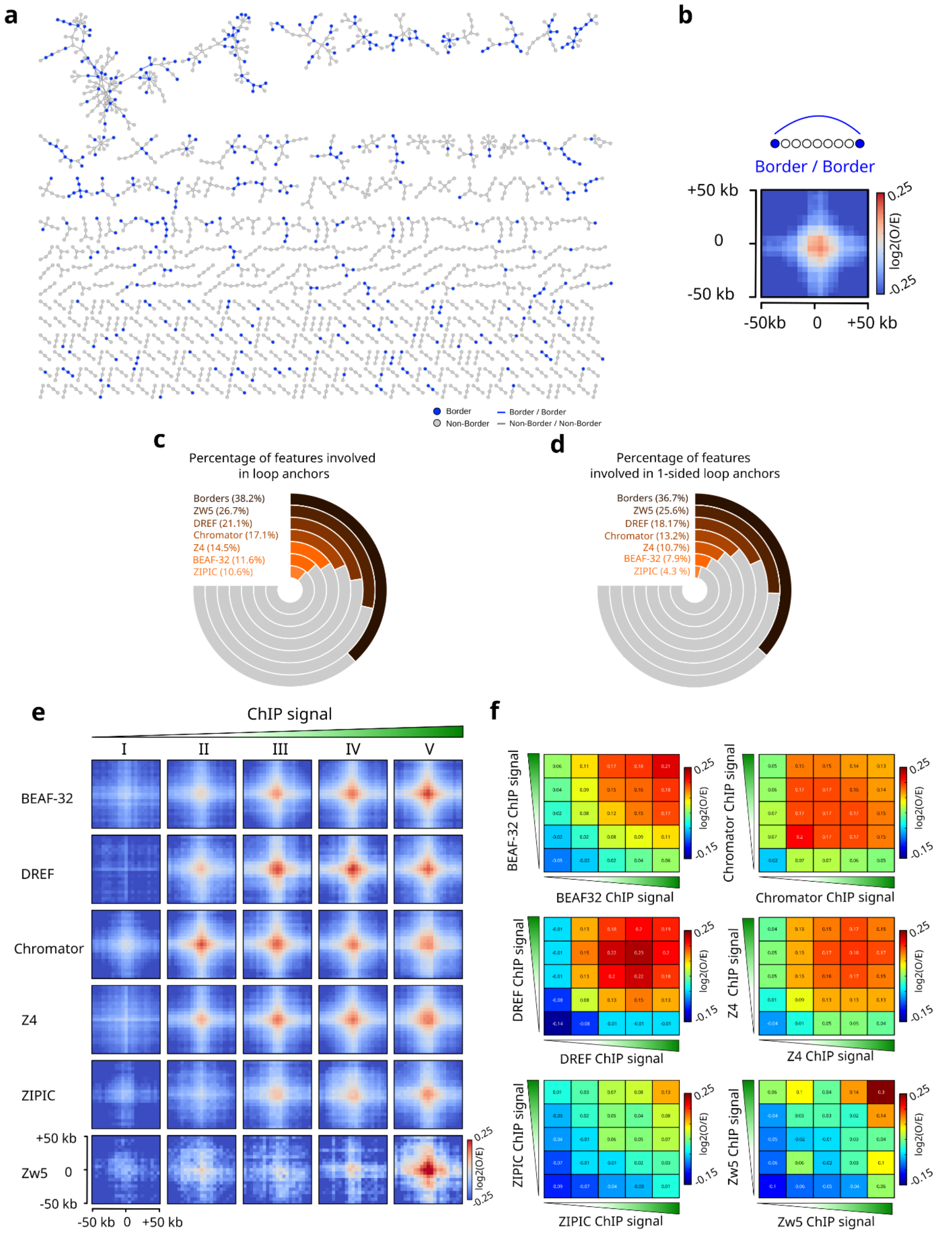

a. Full chromosight chromatin network from Hi-C data at nc14 embryos [1]. Subnetworks containing less than 3 nodes are not represented. **b**. Aggregation Hi-C plots based on Hi-C data for TADs borders called in nc14. **c**. Radial bar chart representing the percentage of features (i.e borders or insulator peaks) involved in loop anchors. Only non-border interactions are considered. **d**. Radial bar chart representing the percentage of features (i.e borders or insulator peaks) involved in 1-sided loop anchors. Only non-border interactions are considered. The majority of features are involved in 1-sided loops. **e**. Aggregation Hi-C plots for each individual protein of the Class I IBP group in nc14 embryos classified by increasing ChIP signal. **f**. log2(O/E) for each individual protein of the Class I IBP group in nc14 ranked by increasing insulator enrichment in nc14 embryos.

Supplementary Figure S3. Supplementary Data complementary to Figure 3.

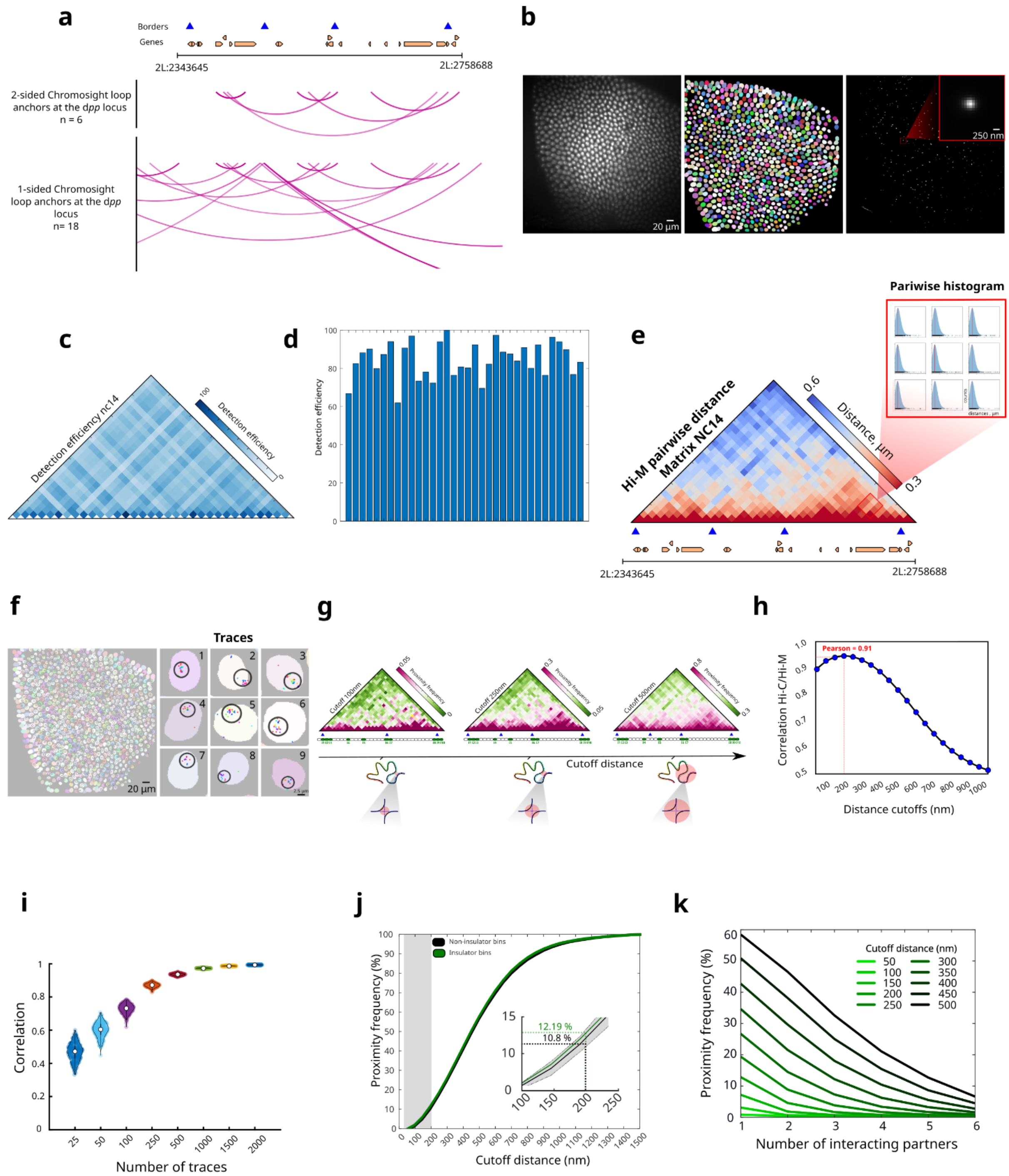

a. Interaction arc diagrams showing called loops from Chromosight at the *Dpp* locus. Top: loops where both anchors are present at the *Dpp* locus are shown. Bottom: loops where at least 1 anchor is found at the *Dpp* locus. **b**. Left: Gray-scale image of DAPI-stained nuclei from nc14 Drosophila embryos. Middle: Segmented DAPI masks after nuclei segmentation. Right: Maximum intensity projection of the fluorescence signal from a single barcode in the same field of view. **c**. Detection efficiency matrices normalized by the most detected barcode pair. Dark blue and white represent high and low detection efficiencies respectively. **d**. Barplot representing the detection efficiency for all barcodes normalized by the most detected barcode. **e**. Hi-M pairwise distance (PWD) matrix for nc14 embryos. The zoom shows the histogram of the pairwise distribution of distances from a subset of a barcode pair. **f**. Example

of segmented barcodes in traces in the same field. The color code indicates different barcodes found in a trace. **g**. Hi-M proximity frequency matrices generated with different cutoff distances for nc14 embryos. From left to right the cutoffs considered are 100, 250, 500 nm. **h**. Pearson correlation coefficient between interpolated Hi-C contact map and Hi-M proximity frequency maps generated with different cutoff distances. The higher correlation is reached with a cutoff of 200 nm. **i**. Violin plots representing the Pearson correlation between and the Hi-M ensemble matrix and matrices generated by sampling subsets of traces by bootstrapping. The Hi-M ensemble matrix was obtained by considering all the traces available. For each condition, 250 bootstrapping cycles were used. **j**. Cumulative proximity frequency versus different cutoff distances for Class I IBP barcodes (green) and for 10 sets of control barcodes (black) for nc14 embryos. For the control, the solid black line represents the mean and the gray shade represents two standard deviations calculated from the variability of controls. **k**. Proximity frequency plotted against the number of interacting partners for Class I IBP barcodes in nc14 embryos, considering different cutoff distances.

Supplementary Figure S4. Supplementary Data complementary to Figure 4.

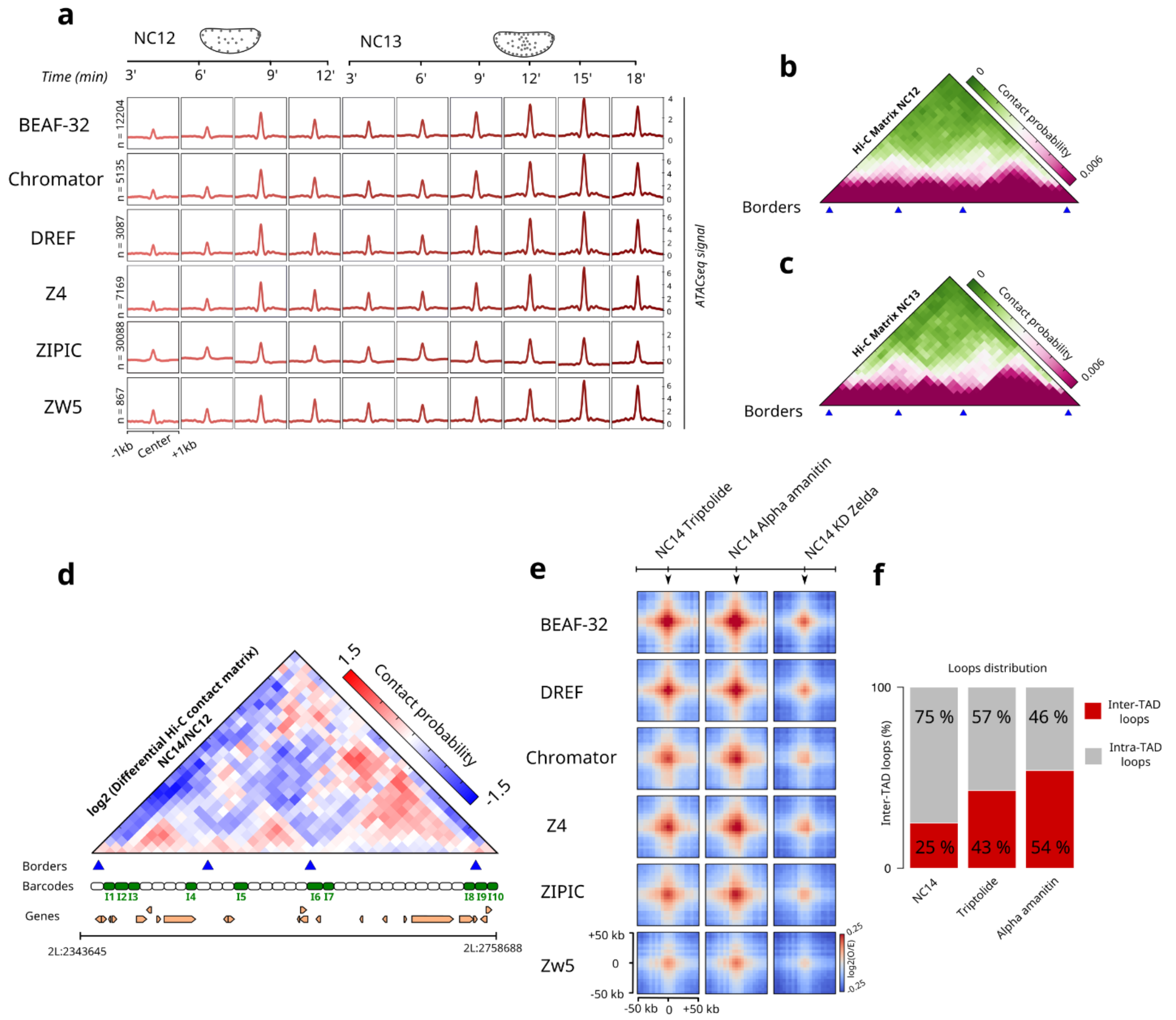

a. Piled ATACseq profiles for each individual Class I IBP across multiple developmental stages (+/- 1kb window). **b**. Nc12 Hi-C matrix from Hug. *et al* [1]. **c**. Nc13 Hi-C matrix from Hug. *et al* [1]. **d**. Log2 differential pairwise Hi-C contact matrix comparing nc14 and nc12. Red and blue indicate higher and lower contacts in nc14 relative to nc12, respectively. **e**. Aggregation Hi-C plots for each individual protein of the non-border Class I IBPs group for different biological conditions and treatments (nc14 triptolide-treated, nc14 alpha-amanitin-treated and nc14 knockdown of Zelda). **h**. Barplot showing the distribution of the percentage of inter-TAD and intra-TAD loops in nc14 wt, nc14 triptolide-treated, nc14 alpha-amanitin-treated.

Supplementary Data

Supplementary_Data_1.xlsx. ChAs Z-Scores and Log2(O/E) values for the different sets of IBPs.

Supplementary_Data_2.xlsx contains the list of sequences of primary Hi-M probes.

Supplementary_Data_3.xlsx. List of primers for library amplification used in this study.

Supplementary_Data_4.xlsx. List of genomic positions of the barcodes used in this study.

Supplementary_Data_5.xlsx contains the list of sequences of imaging (io), adapter oligos and barcodes used in this study.

Supplementary_Data_6.xlsx. List of publicly available data used in this study.

Supplementary References

1. Hug, C. B., Grimaldi, A. G., Kruse, K. & Vaquerizas, J. M. Chromatin Architecture Emerges during Zygotic Genome Activation Independent of Transcription. *Cell* **169,** 216–228.e19 (2017).
2. Batut, P. J., Bing, X. Y., Sisco, Z., Raimundo, J., Levo, M. & Levine, M. S. Genome organization controls transcriptional dynamics during development. *Science* **375,** 566–570 (2022).
