# Supplementary material for: 3D chromatin interactions involving Drosophila insulators are infrequent but preferential and arise before TADs and transcription
Source: Nat Commun. 2023 Oct 21;14:6678. doi: 10.1038/s41467-023-42485-y (PMC10590426; doi:10.1038/s41467-023-42485-y)
Supplement: Supplementary file 3 — Description of Additional Supplementary Files [file 41467_2023_42485_MOESM3_ESM.pdf]

Description of Additional Supplementary Files

File Name: Supplementary Data 1
Description: ChAs Z-Scores and Log2 O/E for the different sets of IBPs.

File Name: Supplementary Data 2
Description: List of sequences of primary Hi-M probes.

File Name: Supplementary Data 3
Description: List of primers for library amplification used in this study.

File Name: Supplementary Data 4
Description: List of genomic positions of the barcodes used in this study.

File Name: Supplementary Data 5
Description: List of sequence of imaging (io), adapter oligos and barcodes used in this study.

File Name: Supplementary Data 6
Description: List of publicly available data used in this study.
